# Supplementary material for: The Transcriptional Profile of Trichophyton rubrum Co-Cultured with Human Keratinocytes Shows New Insights about Gene Modulation by Terbinafine
Source: Pathogens. 2019 Nov 29;8(4):274. doi: 10.3390/pathogens8040274 (PMC6963840; doi:10.3390/pathogens8040274)
Supplement: Supplementary file 1 [file pathogens-08-00274-s001.pdf]

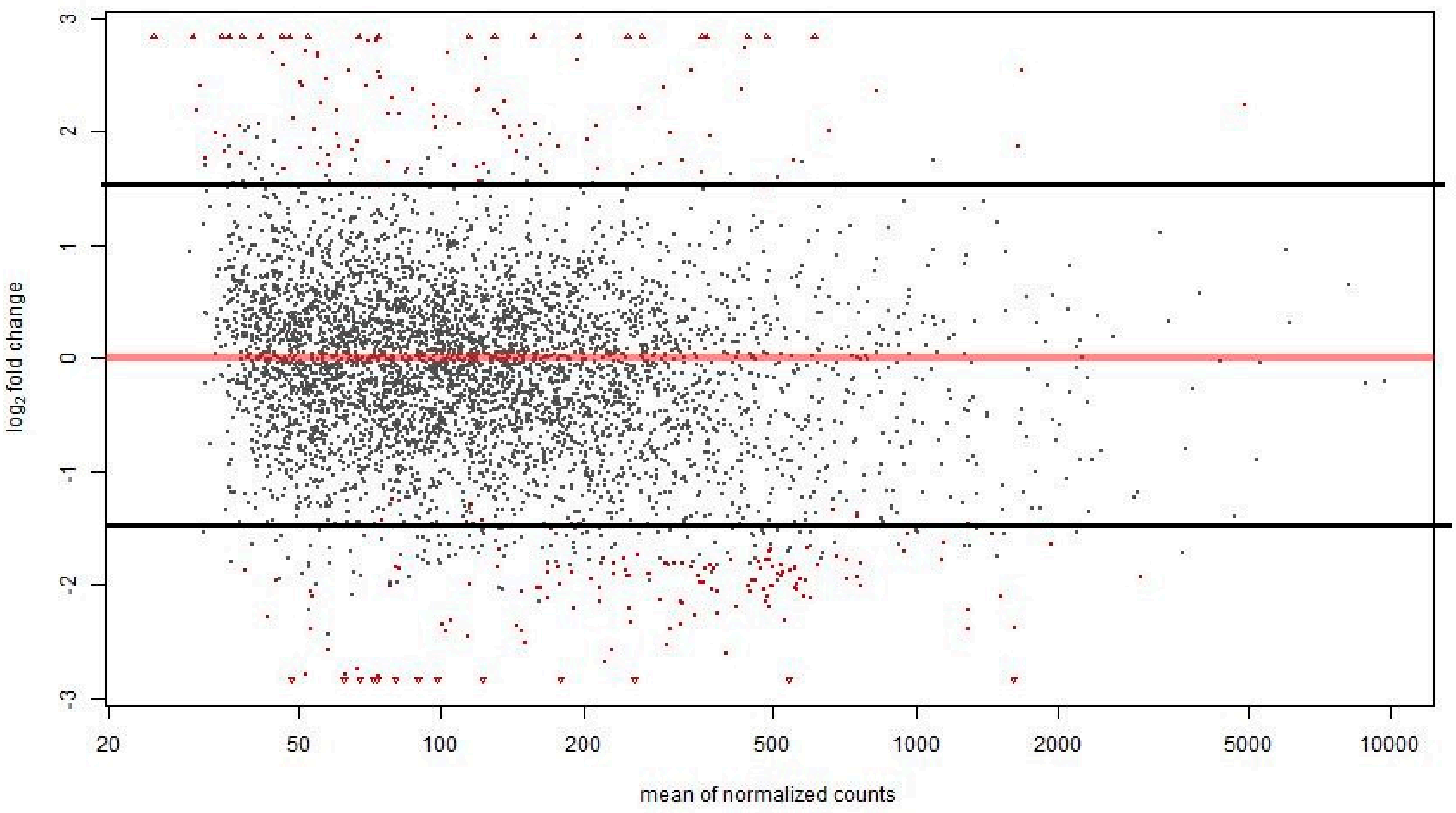

Figure 1. Distribution of differentially expressed genes after 24 h of co-culture. The red points indicate differentially expressed genes.

Table S1. General features of RNA-seq sequences against reference genomes.

			T. rubrum reference genome (CBS 118892)		Human reference genome (HG19)	
**Sample**	**Raw reads**	**High-quality reads**	**Mapped reads**	**Mapped reads (%)**	**Mapped reads**	**Mapped reads (%)**
CO I (PE)	50.412.147	48.758.632	351.062	0.72%	41.752.016	85.63%
CO II (SR)	27.926.550	27.331.638	2.571.234	9.41%	22.298.067	81.58%
CO III (SR)	25.574.109	25.063.089	1.388.085	5.54%	21.400.355	85.39%
COT I (PE)	68.296.564	65.850.177	230.475	0.35%	56.710.172	86.12%
COT II (SR)	26.040.580	25.462.413	02.656.523	10.43%	20.399.609	80.12%
COT III (SR)	23.831.733	23.293.557	02.174.301	09.33%	18.923.004	81.24%
H I (SR)	35.308.490	34.530.495	-	-	30.625.029	88.69%
H II (PE)	61.220.210	58.106.283	-	-	48.611.716	83.66%
H III (PE)	45.385.154	42.869.051	-	-	35.992.855	83.96%
HT I (SR)	32.872.913	32.156.689	-	-	28.490.482	88.60%
HT II (SR)	42.322.033	40.063.232	-	-	33.496.868	83.61%
HT III (SR)	50.914.306	48.502.127	-	-	40.601.130	83.71%

CO I. CO II. CO III: co-culture libraries; COT I. COT II. COT III: co-culture with terbinafine libraries; H I. H II. H III: human keratinocyte libraries; HT I. HT II. HT III: human keratinocyte with terbinafine libraries. The libraries were constructed in triplicate. with I. II and III corresponding to the sample number of each condition. PE: paired-end sequence; SR: single read sequence.

Table S2: Complete list of up-regulated genes in *T. rubrum* after 24 h of co-culture treated with terbinafine.

ID	Log_2 fold change	Gene product name	Orthologous
TERG_00523	5,50	Hypothetical protein	-
TERG_08182	4,79	Hypothetical protein	-
TERG_01636	4,72	Hypothetical protein	Microsporum gypseum CBS 118893 ADP-ribosylglycohydrolase (1715 nt)
TERG_02902	4,64	Hypothetical protein	-
TERG_00540	4,44	Hypothetical protein	Trichophyton verrucosum HKI 0517 oxidoreductase, zinc-binding, putative (1134 nt)
TERG_12029	4,19	Hypothetical protein	-
TERG_02067	4,03	Hypothetical protein	-
TERG_02609	3,93	Sucrase/ferredoxin domain-containing protein	Trichophyton equinum CBS 127.97 actin patches distal protein 1 (950 nt)
TERG_06347	3,93	Hypothetical protein	-
TERG_11747	3,79	Hypothetical protein	-
TERG_08503	3,45	Hypothetical protein	Microsporum canis CBS 113480 secalin (2547 nt)
TERG_08121	3,38	Protein kinase subdomain-containing protein	Microsporum gypseum CBS 118893 protein kinase subdomain-containing protein (891 nt)
TERG_08278	3,33	Serine/threonine protein kinase	Trichophyton tonsurans CBS 112818 serine/threonine protein kinase (2270 nt)
TERG_05843	3,32	Hypothetical protein	Trichophyton equinum CBS 127.97 F-box domain-containing protein (2405 nt)
TERG_08194	3,24	Hypothetical protein	Microsporum canis CBS 113480 serine/threonine protein kinase (2427 nt)

TERG_00197	3,23	Aldose 1-epimerase	Trichophyton verrucosum HKI 0517 aldose 1-epimerase family protein, putative (1631 nt)
TERG_02899	3,17	Hypothetical protein	-
TERG_05111	3,12	Hypothetical protein	-
TERG_03293	3,03	Hypothetical protein	-
TERG_03132	2,91	Hypothetical protein	-
TERG_00959	2,79	Hypothetical protein	Arthroderma benhamiae CBS 112371 RNA binding protein, putative (3143 nt)
TERG_03252	2,78	Hypothetical protein	-
TERG_03304	2,77	Hypothetical protein	Trichophyton verrucosum HKI 0517 AAA family ATPase, putative (2368 nt)
TERG_06402	2,75	Hypothetical protein	Trichophyton verrucosum HKI 0517 Ser/Thr protein phosphatase family protein (921 nt)
TERG_02448	2,74	Hypothetical protein	-
TERG_11932	2,74	Hypothetical protein	-
TERG_02303	2,74	Ankyrin repeat protein	Arthroderma benhamiae CBS 112371 ankyrin repeat protein (5579 nt)
TERG_04951	2,73	Hypothetical protein	Trichophyton equinum CBS 127.97 U-box domain-containing protein (2461 nt)
TERG_06445	2,72	Hypothetical protein	-
TERG_06992	2,69	Pyridine nucleotide-disulfide oxidoreductase	Trichophyton tonsurans CBS 112818 pyridine nucleotide-disulphide oxidoreductase (1785 nt)
TERG_02900	2,69	Hypothetical protein	-
TERG_04234	2,66	Hypothetical protein	Trichophyton verrucosum HKI 0517 hydrophobin, putative (562 nt)
TERG_00583	2,63	Hypothetical protein	-

TERG_08046	2,56	Hypothetical protein	Microsporum gypseum CBS 118893 beta-lactamase (852 nt)
TERG_04721	2,54	Glutamate carboxypeptidase	Trichophyton equinum CBS 127.97 glutamate carboxypeptidase (2412 nt)
TERG_05909	2,51	Hypothetical protein	-
TERG_05239	2,46	DNA polymerase lambda	Trichophyton verrucosum HKI 0517 DNA polymerase POL4, putative (2133 nt)
TERG_01956	2,46	Hypothetical protein	Arthroderma benhamiae CBS 112371 C2H2 finger domain protein, putative (3359 nt)
TERG_06065	2,43	Hypothetical protein	Trichophyton verrucosum HKI 0517 conserved glycine-rich protein (903 nt)
TERG_06207	2,39	Hypothetical protein	Trichophyton verrucosum HKI 0517 proline oxidase PrnD (1941 nt)
TERG_07034	2,39	Hypothetical protein	-
TERG_03861	2,39	C2H2 transcription factor	Trichophyton tonsurans CBS 112818 C2H2 transcription factor (895 nt)
TERG_03443	2,34	Hypothetical protein	Trichophyton equinum CBS 127.97 ankyrin repeat protein (1561 nt)
TERG_03628	2,33	Serine/threonine protein kinase	Trichophyton tonsurans CBS 112818 serine/threonine protein kinase (2187 nt)
TERG_00642	2,32	Hypothetical protein	Trichophyton equinum CBS 127.97 HHE domain-containing protein (552 nt)
TERG_07982	2,32	Hypothetical protein	-
TERG_05469	2,32	Hypothetical protein	-
TERG_12329	2,32	Hypothetical protein	Trichophyton tonsurans CBS 112818 Ku70/Ku80 beta-barrel domain-containing protein (2242 nt)
TERG_06990	2,30	Hypothetical protein	-
TERG_02131	2,29	Hypothetical protein	-
TERG_08693	2,26	Hypothetical protein	Trichophyton equinum CBS 127.97 leptomycin B resistance protein pmd1 (3963 nt)

TERG_00244	2,26	Stomatin family protein	Trichophyton tonsurans CBS 112818 stomatin family protein (1286 nt)
TERG_07035	2,26	Hypothetical protein	-
TERG_08422	2,22	Hypothetical protein	-
TERG_05480	2,20	Hypothetical protein	-
TERG_02644	2,20	Hypothetical protein	-
TERG_11593	2,20	Hypothetical protein	Arthroderma benhamiae CBS 112371 carboxypeptidase Y, putative (1705 nt)
TERG_06239	2,19	Hypothetical protein	-
TERG_06208	2,16	Hypothetical protein	Microsporum gypseum CBS 118893 DUF636 domain-containing protein (639 nt)
TERG_11545	2,15	Hypothetical protein	Trichophyton tonsurans CBS 112818 sugar transporter (2762 nt)
TERG_06106	2,13	Sulfate permease 2	Trichophyton tonsurans CBS 112818 sulfate permease 2 (2623 nt)
TERG_04003	2,13	Phenylacetyl-CoA ligase	Trichophyton tonsurans CBS 112818 AMP dependent CoA ligase (1964 nt)
TERG_06085	2,12	DNA-directed DNA polymerase theta	Trichophyton equinum CBS 127.97 DNA-directed DNA polymerase theta (3180 nt)
TERG_07723	2,08	Oxidoreductase	Trichophyton verrucosum HKI 0517 oxidoreductase, short chain dehydrogenase/reductase family superfamily (870 nt)
TERG_04598	2,07	2-succinylbenzoate-CoA ligase	Arthroderma benhamiae CBS 112371 NRPS-like enzyme, putative (1537 nt)
TERG_08751	2,07	Hypothetical protein	Arthroderma benhamiae CBS 112371 ABC multidrug transporter, putative (3967 nt)
TERG_00575	2,07	Hypothetical protein	Trichophyton verrucosum HKI 0517 F-box domain protein (2021 nt)
TERG_03415	2,06	Serine/threonine protein kinase	Trichophyton tonsurans CBS 112818 serine/threonine protein kinase (4102 nt)

TERG_12614	2,06	Hypothetical protein	Trichophyton verrucosum HKI 0517 succinyl-CoA synthetase beta subunit, putative (2604 nt)
TERG_07983	2,04	Hypothetical protein	-
TERG_12093	2,02	Hypothetical protein	Trichophyton verrucosum HKI 0517 poly(ADP)-ribose polymerase PARP, putative (2610 nt)
TERG_05012	2,02	TPR domain-containing protein	Trichophyton verrucosum HKI 0517 TPR domain protein (1704 nt)
TERG_04103	1,99	Hypothetical protein	-
TERG_08286	1,99	Hypothetical protein	Trichophyton verrucosum HKI 0517 GPI anchored CFEM domain protein (696 nt)
TERG_03789	1,98	Hypothetical protein	Arthroderma benhamiae CBS 112371 glutamine-serine rich protein MS8, putative (887 nt)
TERG_08116	1,97	Von Willebrand factor	-
TERG_03223	1,96	N-acetylglucosamine-6-phosphate deacetylase	Trichophyton tonsurans CBS 112818 n-acetylglucosamine-6-phosphate deacetylase (1349 nt)
TERG_06679	1,95	Hypothetical protein	Arthroderma benhamiae CBS 112371 MFS transporter, putative (1743 nt)
TERG_07859	1,91	Ubiquitin C-terminal hydrolase L3	Trichophyton tonsurans CBS 112818 ubiquitin C-terminal hydrolase L3 (884 nt)
TERG_05497	1,91	Hypothetical protein	Trichophyton equinum CBS 127.97 C6 zinc finger domain-containing protein (1632 nt)
TERG_03064	1,90	Hypothetical protein	Trichophyton equinum CBS 127.97 MCAK-like kinesin (2001 nt)
TERG_02912	1,89	MFS transporter	Arthroderma benhamiae CBS 112371 MFS transporter, putative (1629 nt)
TERG_03381	1,88	DNA damage repair protein Mus42	Trichophyton tonsurans CBS 112818 DNA damage repair protein Mus42 (3520 nt)
TERG_12328	1,88	Hypothetical protein	Trichophyton tonsurans CBS 112818 Ku70/Ku80 beta-barrel domain-containing protein (2242 nt)

TERG_00348	1,87	Galactose-proton symporter	Trichophyton tonsurans CBS 112818 sugar transporter (2762 nt)
TERG_05688	1,84	Hypothetical protein	Trichophyton equinum CBS 127.97 GTP binding protein (2317 nt)
TERG_00394	1,84	Hypothetical protein	Trichophyton verrucosum HKI 0517 LMBR1 domain protein (1809 nt)
TERG_04691	1,84	Hypothetical protein	Trichophyton verrucosum HKI 0517 nuclear division Rft1 protein, putative (1640 nt)
TERG_04973	1,84	Hypothetical protein	Trichophyton tonsurans CBS 112818 beta-ketoacyl synthase (7211 nt)
TERG_00574	1,83	Hypothetical protein	Trichophyton verrucosum HKI 0517 MFS multidrug transporter, putative (1871 nt)
TERG_08480	1,82	Thioredoxin	Trichophyton tonsurans CBS 112818 thioredoxin (1950 nt)
TERG_03200	1,80	DNA repair protein Rhp26/Rad26	Trichophyton tonsurans CBS 112818 DNA repair and recombination protein RAD26 (3815 nt)
TERG_07984	1,78	Hypothetical protein	-
TERG_05626	1,78	Hypothetical protein	Trichophyton equinum CBS 127.97 chitinase (4758 nt)
TERG_05528	1,78	Hypothetical protein	-
TERG_03944	1,77	Hypothetical protein	-
TERG_11666	1,76	Hypothetical protein	Trichophyton equinum CBS 127.97 Ku family DNA helicase (2792 nt)
TERG_03672	1,74	Hypothetical protein	-
TERG_01124	1,74	Hypothetical protein	Trichophyton verrucosum HKI 0517 Ran-interacting protein Mog1, putative (1025 nt)
TERG_08123	1,74	Hypothetical protein	Arthroderma benhamiae CBS 112371 C6 finger domain protein, putative (1558 nt)
TERG_03981	1,73	Hypothetical protein	-
TERG_02742	1,72	Glycosyl hydrolase	Trichophyton tonsurans CBS 112818 glycosyl hydrolase (2705 nt)

TERG_06995	1,72	Metalloreductase	Trichophyton verrucosum HKI 0517 metalloreductase, putative (1917 nt)
TERG_06594	1,71	Hypothetical protein	Trichophyton equinum CBS 127.97 DUF1183 domain-containing protein (867 nt)
TERG_00483	1,70	Hypothetical protein	-
TERG_11594	1,70	Hypothetical protein	Trichophyton tonsurans CBS 112818 serine carboxypeptidase (1689 nt)
TERG_05988	1,68	Hypothetical protein	Trichophyton verrucosum HKI 0517 solid-state culture expressed protein (Aos23), putative (1674 nt)
TERG_02066	1,68	Hypothetical protein	Microsporum gypseum CBS 118893 MGS207 protein (1438 nt)
TERG_01407	1,68	meiotic recombination protein Mre11	Trichophyton equinum CBS 127.97 meiotic recombination protein Mre11 (2939 nt)
TERG_07891	1,68	Hypothetical protein	Trichophyton equinum CBS 127.97 DUF255 domain-containing protein (2611 nt)
TERG_01640	1,67	Hypothetical protein	Trichophyton tonsurans CBS 112818 COPII-coated vesicle protein (1897 nt)
TERG_05490	1,67	Hypothetical protein	#N/D
TERG_07116	1,66	Aldehyde dehydrogenase	Trichophyton tonsurans CBS 112818 aldehyde dehydrogenase (1678 nt)
TERG_05443	1,66	Hypothetical protein	-
TERG_03322	1,65	Hypothetical protein	-
TERG_00149	1,64	Hypothetical protein	-
TERG_01757	1,64	DNA repair protein RAD16	Trichophyton equinum CBS 127.97 DNA repair protein RAD16 (3281 nt)
TERG_08613	1,63	Multidrug resistance protein	Arthroderma benhamiae CBS 112371 ABC multidrug transporter, putative (4048 nt)
TERG_05471	1,63	Hypothetical protein	Microsporum gypseum CBS 118893 activating signal cointegrator 1 complex subunit 3 (5979 nt)

TERG_03294	1,62	Translation initiation protein Sua5	Trichophyton equinum CBS 127.97 SUA5 (1338 nt)
TERG_08058	1,61	Glycosyl hydrolase	Trichophyton verrucosum HKI 0517 alpha-1,2-mannosidase family protein (2923 nt)
TERG_01170	1,60	adenosine deaminase	Trichophyton tonsurans CBS 112818 CECR1 family adenosine deaminase (1986 nt)
TERG_12222	1,58	Hypothetical protein	Trichophyton equinum CBS 127.97 polyketide synthase (7214 nt)
TERG_11610	1,58	Hypothetical protein	-
TERG_02711	1,57	Non-ribosomal peptide synthetase	Trichophyton equinum CBS 127.97 nonribosomal peptide synthase pes1 (10179 nt)
TERG_03830	1,56	Hypothetical protein	-
TERG_04489	1,55	Hypothetical protein	-
TERG_02822	1,54	MFS multidrug transporter	Arthroderma benhamiae CBS 112371 MFS multidrug transporter, putative (1709 nt)
TERG_05575	1,54	MFS multidrug transporter	Trichophyton tonsurans CBS 112818 MFS multidrug transporter (1811 nt)
TERG_07964	1,53	Hypothetical protein	Trichophyton verrucosum HKI 0517 vacuolar endopolyphosphatase, putative (1979 nt)
TERG_01644	1,51	SNF2 family helicase/ATPase	Trichophyton equinum CBS 127.97 SNF2 family helicase/ATPase (4845 nt)

Table S3: Complete list of down-regulated genes in *T. rubrum* after 24 h of co-culture treated with terbinafine.

ID	Log_2 fold change	Gene product name	Orthologous
TERG_01731	-3,68	Hypothetical protein	-
TERG_11886	-3,49	Hypothetical protein	Trichophyton tonsurans CBS 112818 copper radical oxidase (2955 nt)
TERG_06315	-3,32	Hypothetical protein	Arthroderma benhamiae CBS 112371 integral membrane protein (1704 nt)
TERG_00499	-3,32	Hypothetical protein	-
TERG_02811	-3,26	Hypothetical protein	Arthroderma benhamiae CBS 112371 acetyl xylan esterase (Axe1), putative (955 nt)
TERG_01900	-3,25	Aquaglyceroporin	Arthroderma benhamiae CBS 112371 aquaglyceroporin, putative (1058 nt)
TERG_00520	-3,19	Hypothetical protein	-
TERG_03105	-3,16	Hypothetical protein	-
TERG_07234	-2,76	Hypothetical protein	-
TERG_04164	-2,60	Hypothetical protein	-
TERG_07011	-2,58	Hypothetical protein	Arthroderma benhamiae CBS 112371 conserved fungal protein (800 nt)
TERG_12474	-2,48	Hypothetical protein	Trichophyton tonsurans CBS 112818 ABC transporter (2471 nt)
TERG_06276	-2,47	Chromate ion transporter	Trichophyton tonsurans CBS 112818 chromate ion transporter (1906 nt)
TERG_01901	-2,44	Glycerol kinase	Trichophyton equinum CBS 127.97 glycerol kinase (1680 nt)
TERG_00765	-2,38	Hypothetical protein	-

TERG_01406	-2,37	Hypothetical protein	Trichophyton equinum CBS 127.97 phospholipase D (939 nt)
TERG_12475	-2,37	Hypothetical protein	Arthroderma benhamiae CBS 112371 ABC transporter, putative (2336 nt)
TERG_07199	-2,35	Hypothetical protein	-
TERG_05698	-2,33	Beta-lactamase	Trichophyton verrucosum HKI 0517 transesterase (LovD), putative (1428 nt)
TERG_07539	-2,30	Hypothetical protein	Trichophyton tonsurans CBS 112818 multidrug resistance protein (2253 nt)
TERG_11621	-2,27	Hypothetical protein	-
TERG_02161	-2,25	DOC family	Microsporum canis CBS 113480 DOC family protein (456 nt)
TERG_02979	-2,22	Delta(24(24(1)))-sterol reductase	Trichophyton tonsurans CBS 112818 Delta(24(24(1)))-sterol reductase (1551 nt)
TERG_07798	-2,22	Hypothetical protein	-
TERG_02722	-2,21	Hypothetical protein	Trichophyton equinum CBS 127.97 WSC domain containing protein (2371 nt)
TERG_00754	-2,20	Hypothetical protein	-
TERG_04382	-2,15	C-14 sterol reductase	Trichophyton tonsurans CBS 112818 c-14 sterol reductase (1597 nt)
TERG_05808	-2,14	Hypothetical protein	-
TERG_03083	-2,14	3-dehydroquinate synthase	Trichophyton equinum CBS 127.97 pentafunctional AROM polypeptide (4908 nt)
TERG_01703	-2,13	Cytochrome P450 51	Trichophyton equinum CBS 127.97 cytochrome P450 51 (1790 nt)
TERG_08666	-2,13	Hypothetical protein	-
TERG_08545	-2,13	C-4 methylsterol oxidase	Trichophyton equinum CBS 127.97 C-4 methyl sterol oxidase Erg25 (940 nt)
TERG_01676	-2,10	6,7-dimethyl-8-ribityllumazine synthase	Trichophyton tonsurans CBS 112818 6,7-dimethyl-8-ribityllumazine synthase (761 nt)

TERG_04041	-2,10	Sad1/UNC domain-containing protein	Trichophyton verrucosum HKI 0517 Sad1/UNC domain protein (2625 nt)
TERG_07810	-2,09	Hypothetical protein	Trichophyton tonsurans CBS 112818 phospholipase (3533 nt)
TERG_00613	-2,09	Hypothetical protein	-
TERG_04793	-2,04	Cyclin	Trichophyton tonsurans CBS 112818 cyclin (1194 nt)
TERG_06265	-2,03	Hypothetical protein	Trichophyton equinum CBS 127.97 LPS glycosyltransferase (1368 nt)
TERG_08359	-2,01	FAD-dependent monooxygenase	Trichophyton tonsurans CBS 112818 FAD-dependent monooxygenase (1321 nt)
TERG_02842	-2,01	Hypothetical protein	Trichophyton equinum CBS 127.97 6-hydroxy-D-nicotine oxidase (1598 nt)
TERG_03204	-2,00	60S ribosomal protein L7	Trichophyton tonsurans CBS 112818 60S ribosomal protein L7 (1079 nt)
TERG_07797	-1,98	Isoflavone reductase	Trichophyton equinum CBS 127.97 amino acid permease (2474 nt)
TERG_01994	-1,98	Hypothetical protein	Trichophyton equinum CBS 127.97 OPT oligopeptide transporter protein (3035 nt)
TERG_00032	-1,98	Mitochondrial dicarboxylate carrier	Trichophyton tonsurans CBS 112818 mitochondrial dicarboxylate transporter (1162 nt)
TERG_01604	-1,97	60S ribosomal protein L36	Trichophyton tonsurans CBS 112818 60S ribosomal protein L36 (527 nt)
TERG_03148	-1,95	Hypothetical protein	Trichophyton equinum CBS 127.97 molybdenum cofactor sulfurase (1551 nt)
TERG_05236	-1,94	60S ribosomal protein L35	Trichophyton tonsurans CBS 112818 60S ribosomal protein L35 (1143 nt)
TERG_01399	-1,93	Hypothetical protein	-
TERG_06755	-1,91	C-8 sterol isomerase	Trichophyton verrucosum HKI 0517 C-8 sterol isomerase (Erg-1), putative (628 nt)
TERG_02542	-1,91	Integral membrane protein	Trichophyton verrucosum HKI 0517 integral membrane protein Pth11-like, putative (1773 nt)
TERG_02188	-1,91	Mechanosensitive ion channel family protein	Trichophyton tonsurans CBS 112818 mechanosensitive ion channel family protein (3018 nt)

TERG_02160	-1,91	Hypothetical protein	-
TERG_00235	-1,89	Hypothetical protein	-
TERG_07154	-1,89	Hypothetical protein	Trichophyton verrucosum HKI 0517 mitochondrial chaperone Frataxin, putative (705 nt)
TERG_01178	-1,88	Hypothetical protein	-
TERG_08108	-1,88	40S ribosomal protein S22	Trichophyton tonsurans CBS 112818 30S ribosomal protein S8 (737 nt)
TERG_01786	-1,86	DENN domain-containing protein	Trichophyton tonsurans CBS 112818 DENN domain-containing protein (2886 nt)
TERG_00024	-1,86	ADP-ribosylation factor	Trichophyton tonsurans CBS 112818 ADP-ribosylation factor (845 nt)
TERG_05524	-1,84	Phospho-2-dehydro-3-deoxyheptonate aldolase	Trichophyton tonsurans CBS 112818 phospho-2-dehydro-3-deoxyheptonate aldolase (1765 nt)
TERG_08360	-1,83	Metallo-beta-lactamase	Trichophyton equinum CBS 127.97 metallo-beta-lactamase superfamily protein (1062 nt)
TERG_07232	-1,82	Sodium transport ATPase	Trichophyton equinum CBS 127.97 calcium-transporting ATPase (3453 nt)
TERG_12533	-1,82	Hypothetical protein	-
TERG_03697	-1,82	Hypothetical protein	-
TERG_07347	-1,81	Hypothetical protein	Trichophyton verrucosum HKI 0517 La domain family (2776 nt)
TERG_00615	-1,81	Hypothetical protein	Arthroderma benhamiae CBS 112371 HLH transcription factor, putative (3219 nt)
TERG_04862	-1,81	C6 sexual development transcription factor NosA	Arthroderma benhamiae CBS 112371 C6 transcription factor RosA (2180 nt)
TERG_02973	-1,81	Morphogenesis protein	Trichophyton verrucosum HKI 0517 morphogenesis protein (Msb1), putative (3063 nt)
TERG_02472	-1,76	Small Rho-type GTPase	Trichophyton equinum CBS 127.97 rho3 protein (1071 nt)

TERG_06611	-1,76	Hypothetical protein	-
TERG_01088	-1,75	3,4-dihydroxy-2-butanone 4-phosphate synthase	Trichophyton equinum CBS 127.97 3,4-dihydroxy-2-butanone 4-phosphate synthase (846 nt)
TERG_07002	-1,75	60S ribosomal protein L17	Trichophyton equinum CBS 127.97 60S ribosomal protein L17 (926 nt)
TERG_02237	-1,75	40S ribosomal protein S28	Trichophyton tonsurans CBS 112818 30S ribosomal protein S28e (482 nt)
TERG_05799	-1,74	Alcohol dehydrogenase	Arthroderma benhamiae CBS 112371 zinc-containing alcohol dehydrogenase, putative (1458 nt)
TERG_02694	-1,74	Sulfite efflux pump	Trichophyton equinum CBS 127.97 sulphite efflux pump protein (1653 nt)
TERG_11582	-1,74	Hypothetical protein	-
TERG_05518	-1,73	Short chain dehydrogenase	Arthroderma benhamiae CBS 112371 oxidoreductase, short chain dehydrogenase/reductase family (1062 nt)
TERG_01102	-1,73	60S ribosomal protein L8	Trichophyton tonsurans CBS 112818 ribosomal protein L7a (1212 nt)
TERG_00633	-1,73	40S ribosomal protein S16	Trichophyton tonsurans CBS 112818 40S ribosomal protein S9 (779 nt)
TERG_02162	-1,71	Hypothetical protein	-
TERG_06317	-1,71	Phosducin	Trichophyton verrucosum HKI 0517 phosducin, putative (1031 nt)
TERG_07099	-1,71	Hypothetical protein	-
TERG_07565	-1,70	Hypothetical protein	-
TERG_03528	-1,69	Cytochrome C	Trichophyton equinum CBS 127.97 cytochrome c (618 nt)
TERG_05685	-1,69	60S ribosomal protein L13	Trichophyton tonsurans CBS 112818 60S ribosomal protein L13 (1133 nt)
TERG_04558	-1,69	Nucleoside-diphosphate kinase	Trichophyton tonsurans CBS 112818 nucleoside diphosphate kinase (884 nt)

TERG_00791	-1,68	Hypothetical protein	Trichophyton verrucosum HKI 0517 developmental regulatory protein WetA (2082 nt)
TERG_01494	-1,67	Hypothetical protein	-
TERG_02377	-1,67	40S ribosomal protein S9	Trichophyton tonsurans CBS 112818 ribosomal protein S9 (983 nt)
TERG_07071	-1,67	2-isopropylmalate synthase	Trichophyton tonsurans CBS 112818 2-isopropylmalate synthase (2130 nt)
TERG_07775	-1,67	Hypothetical protein	-
TERG_00517	-1,66	Hypothetical protein	-
TERG_04174	-1,66	Hypothetical protein	Trichophyton equinum CBS 127.97 citrinin biosynthesis oxydoreductase CtnB (795 nt)
TERG_07083	-1,65	Hypothetical protein	-
TERG_04175	-1,65	Hypothetical protein	-
TERG_08581	-1,65	F-box protein	Trichophyton tonsurans CBS 112818 F-box protein (1757 nt)
TERG_05717	-1,65	Squalene epoxidase	Trichophyton equinum CBS 127.97 squalene epoxidase (1588 nt)
TERG_00462	-1,65	40S ribosomal protein S3	Trichophyton equinum CBS 127.97 40S ribosomal protein S3 (1080 nt)
TERG_07303	-1,65	TIM complex component Tim54	Trichophyton tonsurans CBS 112818 TIM complex componenet Tim54 (1523 nt)
TERG_06172	-1,65	60S ribosomal protein L12	Trichophyton equinum CBS 127.97 60S ribosomal protein L12 (922 nt)
TERG_11530	-1,65	Hypothetical protein	-
TERG_01344	-1,64	60S ribosomal protein L22	Trichophyton tonsurans CBS 112818 60S ribosomal protein L22 (699 nt)
TERG_05172	-1,63	Alternative NADH-dehydrogenase	Arthroderma benhamiae CBS 112371 pyridine nucleotide-disulphide oxidoreductase family protein (2115 nt)
TERG_12382	-1,63	Hypothetical protein	Trichophyton tonsurans CBS 112818 50S ribosomal protein L34e (1083 nt)

TERG_04282	-1,63	Hypothetical protein	Trichophyton tonsurans CBS 112818 GPI ethanolamine phosphate transferase (717 nt)
TERG_01599	-1,63	Hypothetical protein	-
TERG_12392	-1,62	Hypothetical protein	-
TERG_01004	-1,62	Extracellular sialidase/neuraminidase	Trichophyton equinum CBS 127.97 extracellular sialidase/neuraminidase (1226 nt)
TERG_02545	-1,62	MFS monocarboxylate transporter	Trichophyton equinum CBS 127.97 MFS monocarboxylate transporter (1461 nt)
TERG_12510	-1,62	Hypothetical protein	-
TERG_08991	-1,61	Hypothetical protein	Microsporum gypseum CBS 118893 ATPase 2 nuclear control (2465 nt)
TERG_06330	-1,61	Hypothetical protein	Trichophyton equinum CBS 127.97 C6 zinc finger domain containing protein (2768 nt)
TERG_04839	-1,61	Hypothetical protein	Microsporum canis CBS 113480 developmental regulator flbA (2087 nt)
TERG_02373	-1,61	Hypothetical protein	Microsporum gypseum CBS 118893 transmembrane protein 165 (1699 nt)
TERG_02372	-1,61	Hypothetical protein	-
TERG_03463	-1,60	Hypothetical protein	-
TERG_01562	-1,59	60S ribosomal protein L11	Trichophyton equinum CBS 127.97 60S ribosomal protein (764 nt)
TERG_07799	-1,58	Fatty acid elongase	Trichophyton equinum CBS 127.97 fatty acids protein 3 elongation (1286 nt)
TERG_06158	-1,58	60S ribosomal protein L29	Trichophyton tonsurans CBS 112818 60S ribosomal protein L29 (539 nt)
TERG_02397	-1,58	60S ribosomal protein L37	Trichophyton equinum CBS 127.97 ribosomal protein L37 (1143 nt)
TERG_04899	-1,58	Ubiquitin fusion protein	Trichophyton tonsurans CBS 112818 ubiquitin/60S ribosomal protein L40 fusion (627 nt)

TERG_07588	-1,58	5-methyltetrahydropteroyltriglutamate-homocysteine methyltransferase	Arthroderma benhamiae CBS 112371 Methionine synthase, vitamin-B12 independent, putative (2459 nt)
TERG_05788	-1,58	60S ribosomal protein L20	Trichophyton tonsurans CBS 112818 60S ribosomal protein L18A (870 nt)
TERG_04906	-1,57	Cytochrome P450 61	Arthroderma benhamiae CBS 112371 Cytochrome P450 61 (2707 nt)
TERG_02861	-1,57	40S ribosomal protein S23	Trichophyton tonsurans CBS 112818 ribosomal protein S12 (572 nt)
TERG_07553	-1,57	C2H2 finger domain-containing protein	Trichophyton verrucosum HKI 0517 C2H2 transcription factor Crz1, putative (2354 nt)
TERG_04158	-1,57	Hypothetical protein	Arthroderma benhamiae CBS 112371 ARS binding protein Abp2, putative (2510 nt)
TERG_00165	-1,57	Hypothetical protein	#N/D
TERG_11529	-1,56	Hypothetical protein	#N/D
TERG_01360	-1,55	Acetate regulatory DNA binding protein	Trichophyton verrucosum HKI 0517 C6 transcription factor, putative (3005 nt)
TERG_08133	-1,55	40S ribosomal protein S25	Trichophyton equinum CBS 127.97 40S ribosomal protein S25 (1042 nt)
TERG_02493	-1,55	Hypothetical protein	Trichophyton verrucosum HKI 0517 glyoxalase family protein (2516 nt)
TERG_02023	-1,54	Extracellular matrix protein	Trichophyton verrucosum HKI 0517 extracellular matrix protein, putative (780 nt)
TERG_04143	-1,54	Cytochrome c oxidase subunit V	Trichophyton tonsurans CBS 112818 Cytochrome c oxidase polypeptide V (898 nt)
TERG_07233	-1,53	Hypothetical protein	Trichophyton equinum CBS 127.97 acriflavine sensitivity control protein acr-2 (1801 nt)
TERG_02166	-1,53	Oxidoreductase	Trichophyton equinum CBS 127.97 oxidoreductase (2130 nt)
TERG_08771	-1,53	Hypothetical protein	Trichophyton tonsurans CBS 112818 extracellular serine-threonine rich protein (2338 nt)

TERG_01537	-1,53	Hypothetical protein	#N/D
TERG_06236	-1,53	40S ribosomal protein S19	Trichophyton equinum CBS 127.97 40S ribosomal protein S19 (1012 nt)
TERG_06824	-1,53	60S ribosomal protein L23	Trichophyton tonsurans CBS 112818 alkaline serine protease (819 nt)
TERG_04775	-1,52	Protein kinase subdomain-containing protein	Arthroderma benhamiae CBS 112371 beta-N-hexosaminidase, putative (1851 nt)
TERG_08869	-1,52	Hypothetical protein	#N/D
TERG_04871	-1,51	C2H2 transcription factor	Trichophyton tonsurans CBS 112818 C2H2 transcription factor (1011 nt)
TERG_06806	-1,51	Hypothetical protein	#N/D
TERG_04647	-1,50	60S ribosomal protein L18	Trichophyton equinum CBS 127.97 60S ribosomal protein L18 (865 nt)
TERG_01550	-1,50	60S ribosomal protein L38	Trichophyton tonsurans CBS 112818 60S ribosomal protein L38 (469 nt)
TERG_05032	-1,50	Hypothetical protein	Trichophyton equinum CBS 127.97 C6 transcription factor (2233 nt)

Table S4: Primers used for qPCR analysis

ID	Gene product name	Primer sequence (5'- 3')	Fragment	Reference
TERG_02694	Sulfite efflux pump	F: GTCATCTACTTCCAGCGTCTTG<br>R: GTCTTGGGAAAACACGCTTG	145 bp	This paper
TERG_08613	ABC transporter	F: AGGAGCAAATCAAGGAACGG<br>R: GGATAAAAGGAAACCACGCTG	119 bp	This paper
TERG_03223	N-Acetylglucosamine-6-phosphate deacetylase	F: CCGTCGATTACCAGAGAAGTTC<br>R: CTTGCCAGAGATAGAGTCAACC	117 bp	This paper
TERG_08480	Thioredoxin	F: TCTCACCTCTACCTAACTCAGC<br>R: AGATAAGAGATGTGCCCGTTG	124 bp	This paper
TERG_01703	Cytochrome p450 51	F: AACAGCTACGCTAGCCAGAC<br>R: AATAGTCAGTTGGCGGCACA	77 bp	This paper
TERG_03415	Serine/threonine protein kinase	F: AACCAAACCAATATGCGGCG<br>R: ACCATCGACGAGTATCGCAC	94 bp	This paper
TERG_07904	Tubulin beta chain	F:AACATGATGGCTGCCACTGA<br>R:AAGATGGCAGAGCAGGTAAGGT	253 bp	Jacob et al., 2012
TERG_08193	18S	F:CGCTGGCTTCTTAGAGGGACTAT<br>R:TGCCTCAAACTTCCATCGACTT	51 bp	Bitencourt et al., 2013
TERG_06106	Sulfate permease 2	F: CTATTCTCCTGTTCCGCCTTG<br>R: TGCCATACTTTCCGTCATTATCC	108 bp	This paper
TERG_00348	Galactose-proton symporter	F: GAGATCGCATCGACCTCCAG<br>R: GATCCCGTACTTGGTCGCAA	95 bp	This paper
TERG_02822	MFS multidrug transporter	F: TTTGTCGCATTTGTCGTTGG<br>R: AACTGCTCTGCGTTGGATAG	150 bp	This paper
TERG_02609	Sucrase/ferredoxin domain-containing protein	F: GCCCTTGTAGTGCCTGAGAG<br>R: GTTGTCACATCCGTCTCCGT	96 bp	This paper
TERG_02542	Integral membrane protein	F: TTCGCAGTATCCAACCATCC<br>R: CATCAACGCCAAAGCTCTTC	116 bp	This paper
TERG_05698	Beta-lactamase	F: TGATTTGGGCGGTATTGGCT<br>R: TCGCAACACGTTCTCACCAT	91 bp	This paper

TERG_05717	Squalene epoxidase	F: CCGCTTTATCATGCGCCTTC<br>R: TGGTAGAAACGGCCTTGGTC	80 bp	This paper
TERG_08058	Glycosyl Hydrolase	F: ATACCTTCCGATGCCTAAACAG<br>R: GAACCCTTCCGTTTGCATTG	141 bp	This paper
TERG_03861	Transcription factor C_2H_2	F: ATATTCGCCGTCTTCACAGG<br>R: ATAATGAGAGTGCTGGTGGAAG	97 bp	This paper
